# Supplementary material for: Extremely stable graphene electrodes doped with macromolecular acid
Source: Nat Commun. 2018 May 23;9:2037. doi: 10.1038/s41467-018-04385-4 (PMC5966423; doi:10.1038/s41467-018-04385-4)
Supplement: Supplementary file 1 — Supplementary Information [file 41467_2018_4385_MOESM1_ESM.pdf]

Supplementary information

Extremely stable graphene electrodes doped with macromolecular acid

Kwon et al.

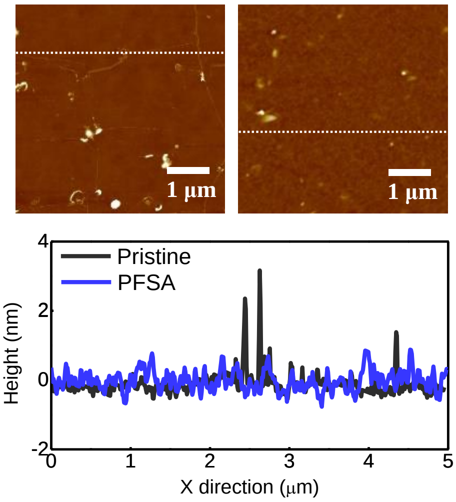

Supplementary Figure 1 | Surface morphology. Atomic force microscopy topographic images of pristine and PFSA-doped graphene (top) and surface heights on the cross section (bottom).

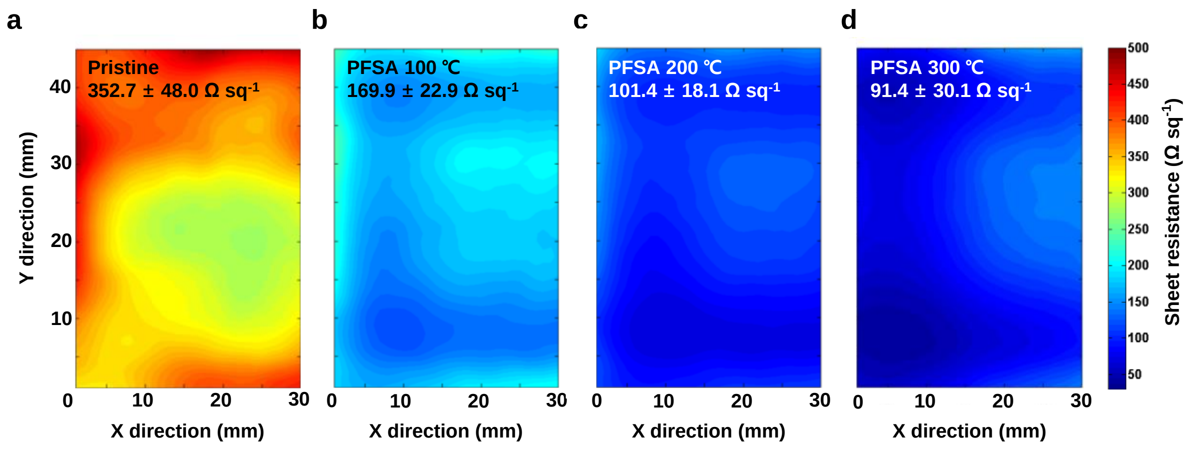

Supplementary Figure 2 | Large-area spatial (30 mm × 45 mm) R_{sh} mapping (a) pristine, and PFSA-doped graphene followed by thermal annealing at (b) 100 °C, (c) 200 °C, (d) 300 °C.

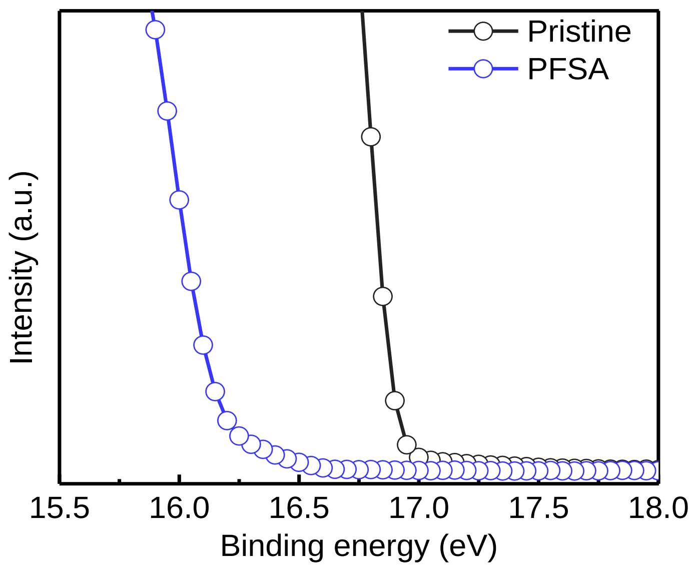

Supplementary Figure 3 | WF change of pristine, and PFSA-doped graphene. Ultraviolet photoelectron spectroscopy spectra of pristine (black) and PFSA-doped graphene (blue).

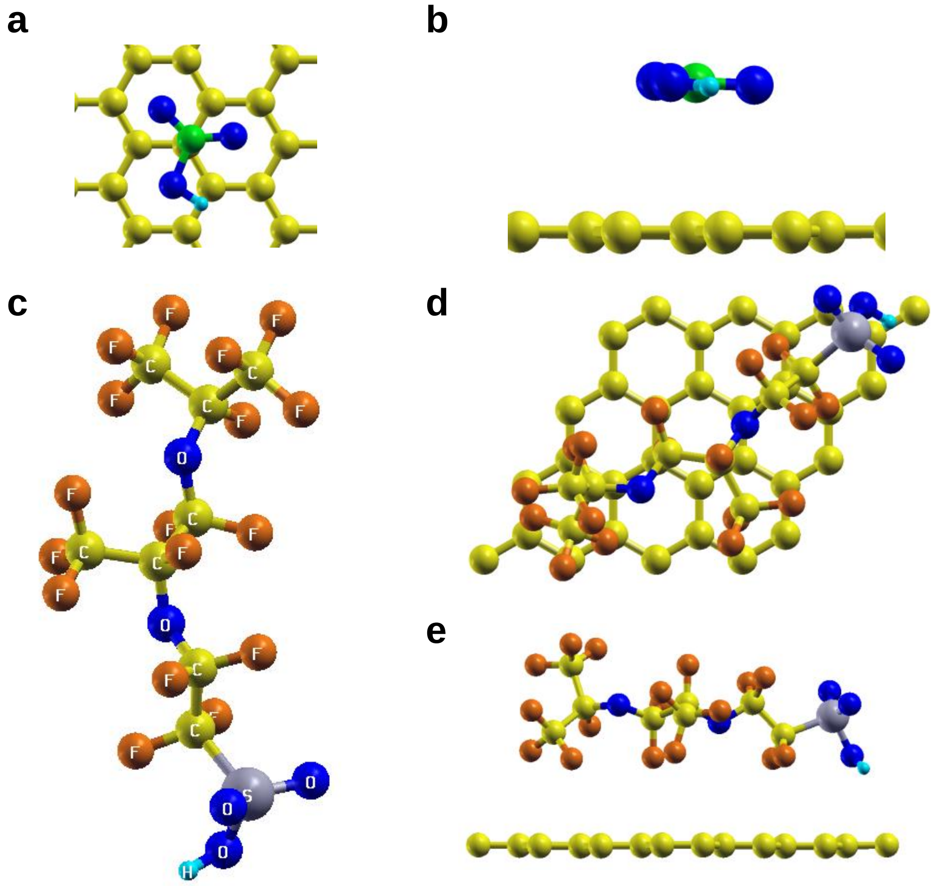

Supplementary Figure 4 | Molecular configuration of p-doped graphene. (a) Top view, and (b) side view of the most favorable configuration of HNO_3-doped graphene. (c) Chemical structure of the simplest PFSA molecule (i.e., $(CF_3)_2CF\text{-}O\text{-}CF_2\text{-}(CF_3)CF\text{-}O\text{-}CF_2CF_2SO_3H$). (d) Top view, and (e) side view of the most favorable configuration of the simplest PFSA molecule. Spheres: blue = oxygen, green = nitrogen, silver = sulfur, cyan = hydrogen, orange = fluorine, yellow = carbon.

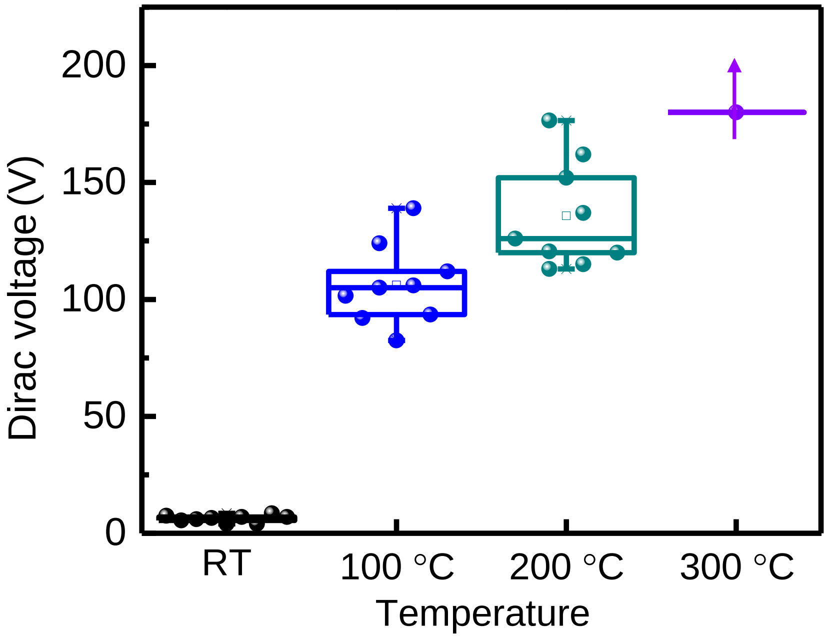

Supplementary Figure 5 | Dirac voltage shift of PFSA-doped graphene. Dirac voltage of pristine graphene (black), PFSA-doped graphene annealed at various T_a. Each box chart includes minimum, lower quartile (lower horizontal line), median (middle horizontal line), mean (hollow square), upper quartile (upper horizontal line), maximum, and discrete data.

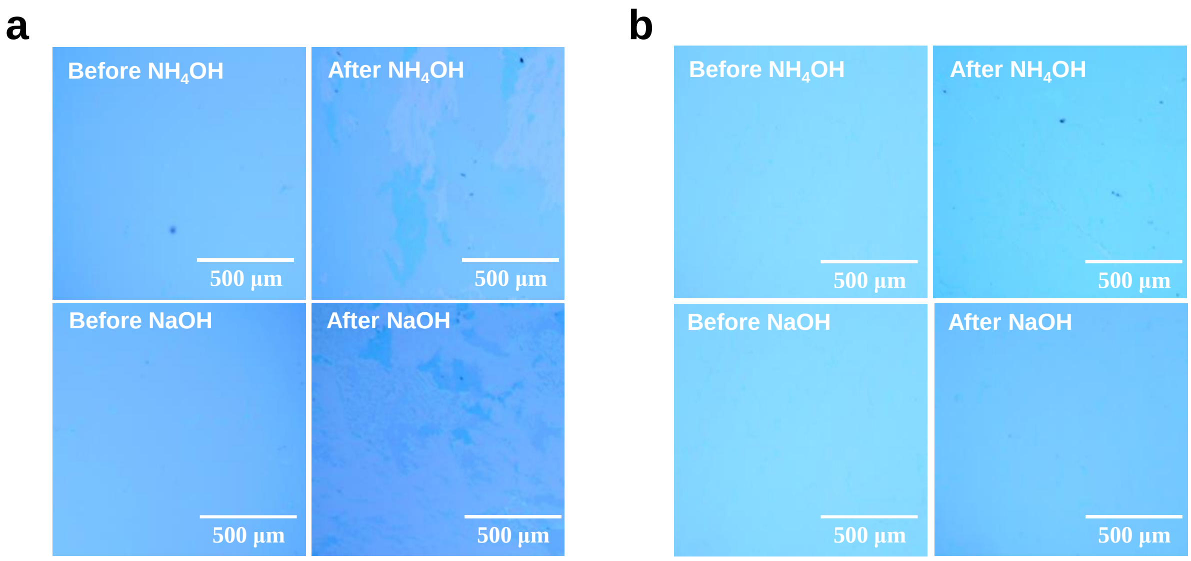

Supplementary Figure 6 | Doping stability against base chemicals. Optical microscopy image of (a) HNO_3-doped graphene, and (b) PFSA-doped graphene (left: before NH_4OH, and NaOH treatment, right: after NH_4OH, and NaOH treatment).

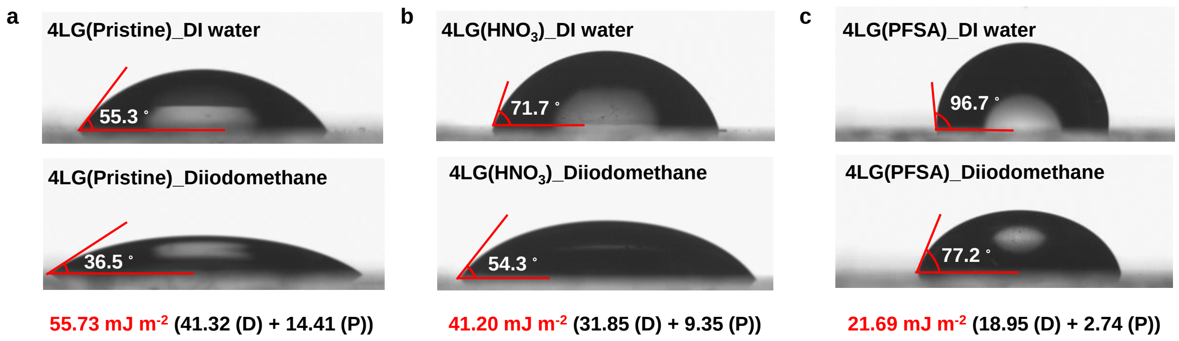

Supplementary Figure 7 | Surface energy calculation. Water and diiodomethane contact angle on (a) pristine, (b) HNO_3-, and (c) PFSA-doped 4LG.

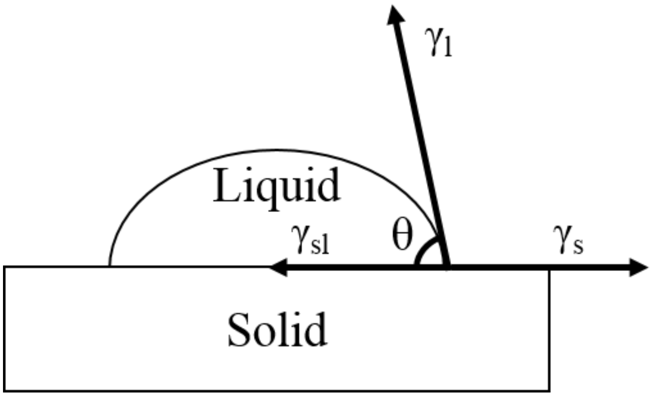

Supplementary Figure 8 | Contact angle and surface tension of a drop deposited on the solid surface.

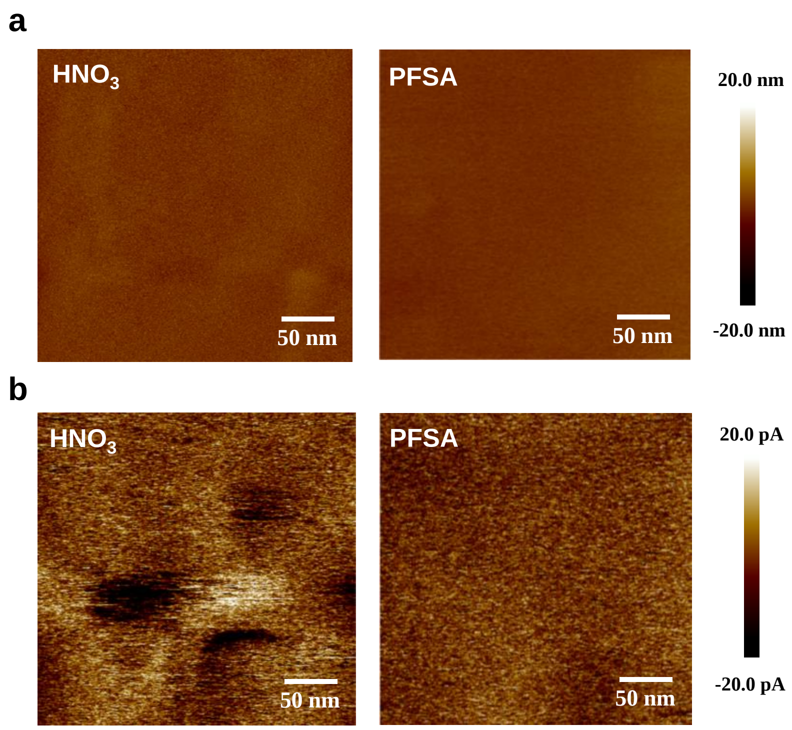

Supplementary Figure 9 | Conductive atomic force microscopy. (a) Surface topography, and (b) electrical current mapping of HNO_3-doped graphene (left), and PFSA-doped graphene (right).

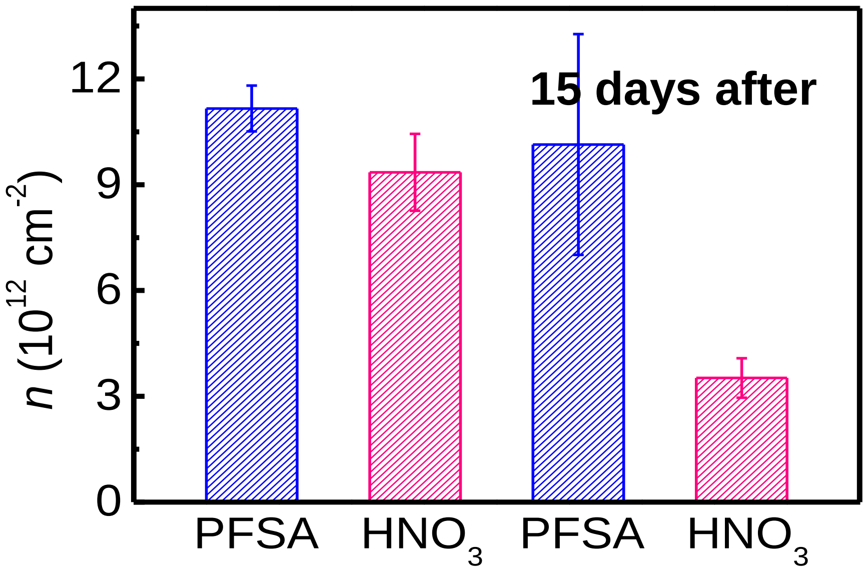

Supplementary Figure 10 | Ambient stability of p-doped graphene. Hole concentration change of PFSA- doped and HNO_3-doped graphene after 15 days calculated using Raman spectroscopy results. The error bars represents the standard deviation of multiple measurement results.

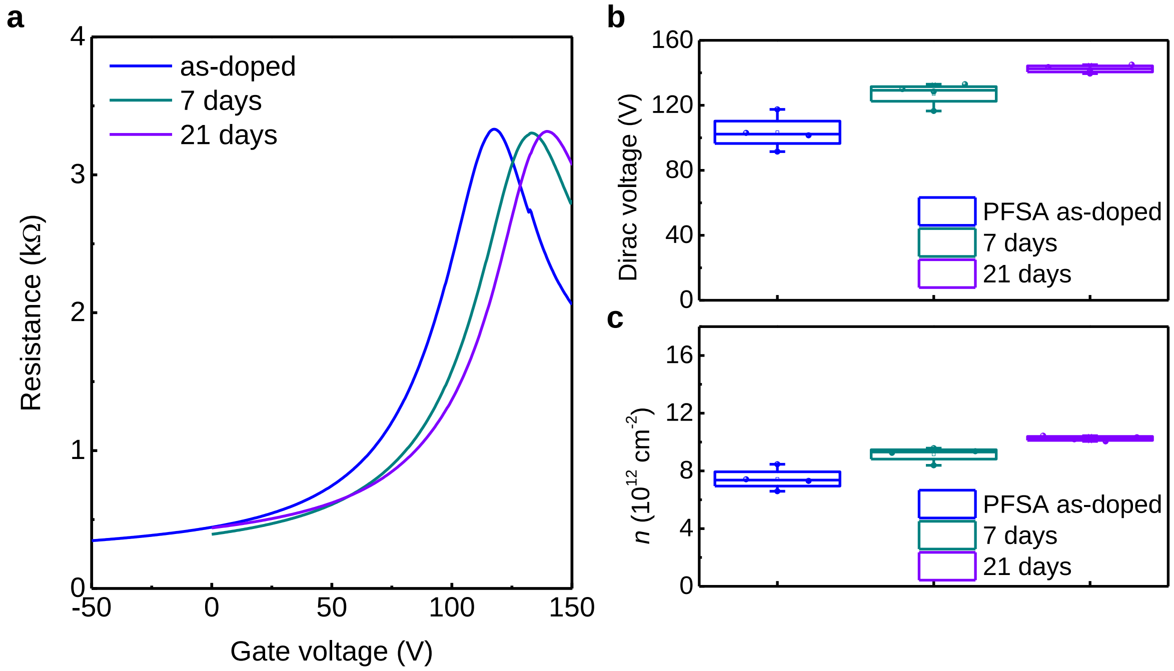

Supplementary Figure 11 | Hole concentration change of PFSA-doped graphene calculated by electrical characteristics of FETs. (a) Current versus voltage characteristics, (b) statistics of Dirac voltage, and (c) hole concentrations of as-doped (blue) and after ambient exposure for 7 days (dark cyan), and 21 days (violet). Each box chart includes minimum, lower quartile (lower horizontal line), median (middle horizontal line), mean (hollow square), upper quartile (upper horizontal line), maximum, and discrete data.

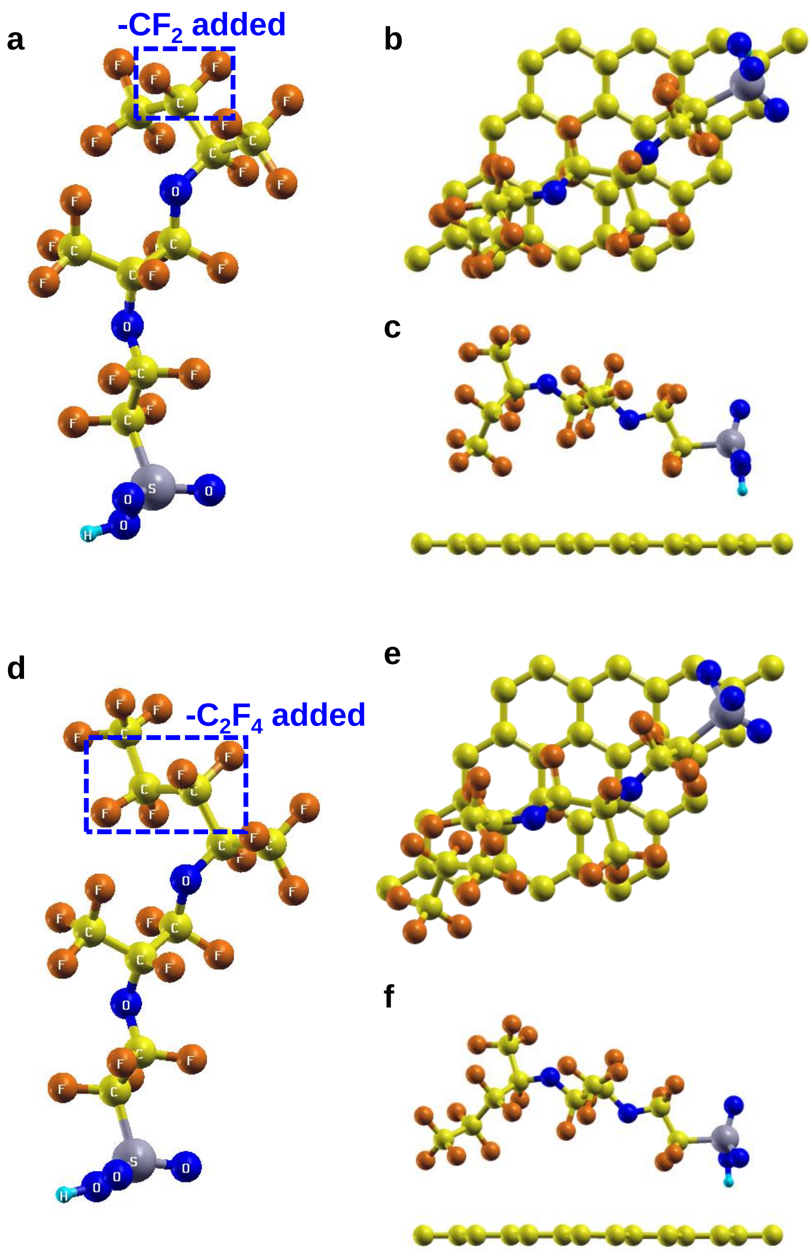

Supplementary Figure 12 | Molecular configuration according to fluorocarbon (-CF_2) addition to PFSA. (a) Chemical structure of the simplest PFSA-CF_2 (*i.e.*, $CF_3(CF_2)CF_3CF$-O-CF_2-(CF_3)CF-O-$CF_2CF_2SO_3H$). (b) Top view, and (c) side view of the most favorable configuration of the simplest PFSA-CF_2, (d) Chemical structure of the simplest PFSA-C_2F_4 (*i.e.*, $CF_3(CF_2)_2CF_3CF$-O-CF_2-(CF_3)CF-O-$CF_2CF_2SO_3H$). (e) Top view, and (f) side view of the most favorable configuration of the simplest PFSA-C_2F_4. Spheres: blue = oxygen, green = nitrogen, silver = sulfur, cyan = hydrogen, orange = fluorine, yellow = carbon.

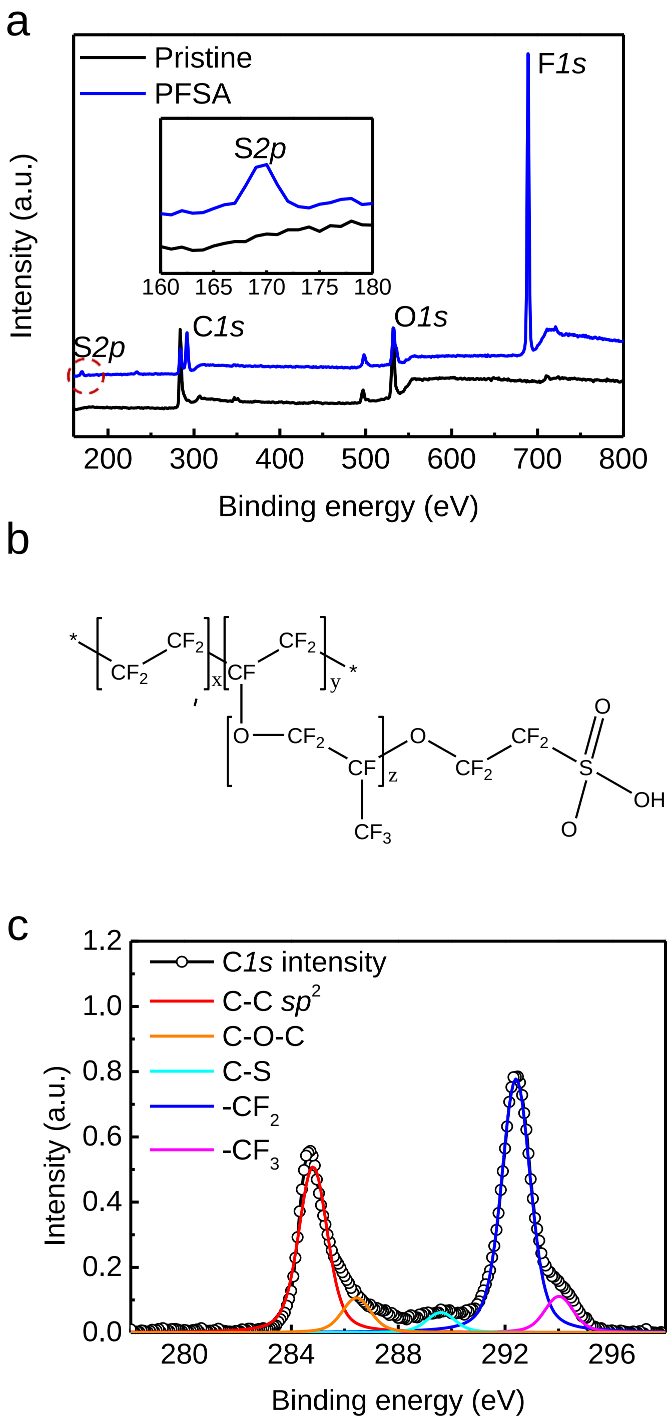

Supplementary Figure 13 | X-ray photoelectron spectroscopy (XPS). (a) Survey spectra of pristine and PFSA-doped graphene. (Inset: enlarged S2*p* peak in survey spectrum of PFSA-doped graphene). (b) Chemical structure of PFSA. (c) Deconvoluted XPS C1*s* spectra of PFSA-doped graphene.

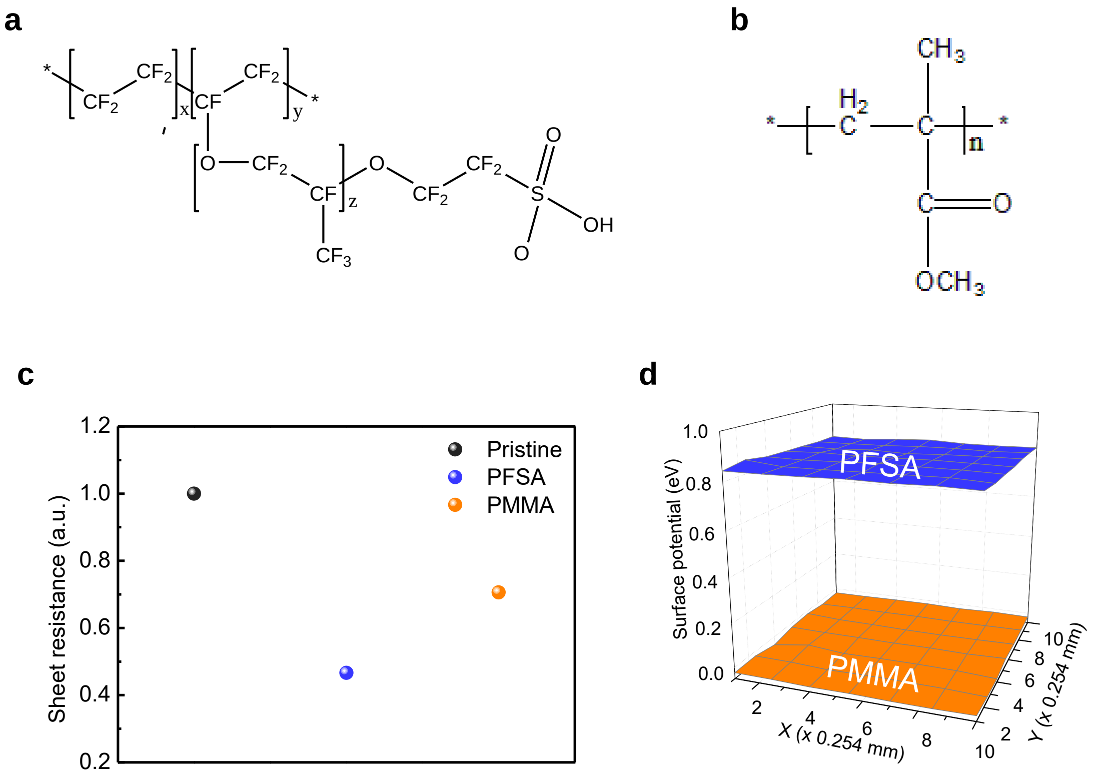

Supplementary Figure 14 | Doping effect of polymers on graphene. Chemical structure of (a) PFSA, and (b) PMMA. (c) R_{sh}, and (d) WF changes of graphene induced by PFSA, and PMMA.

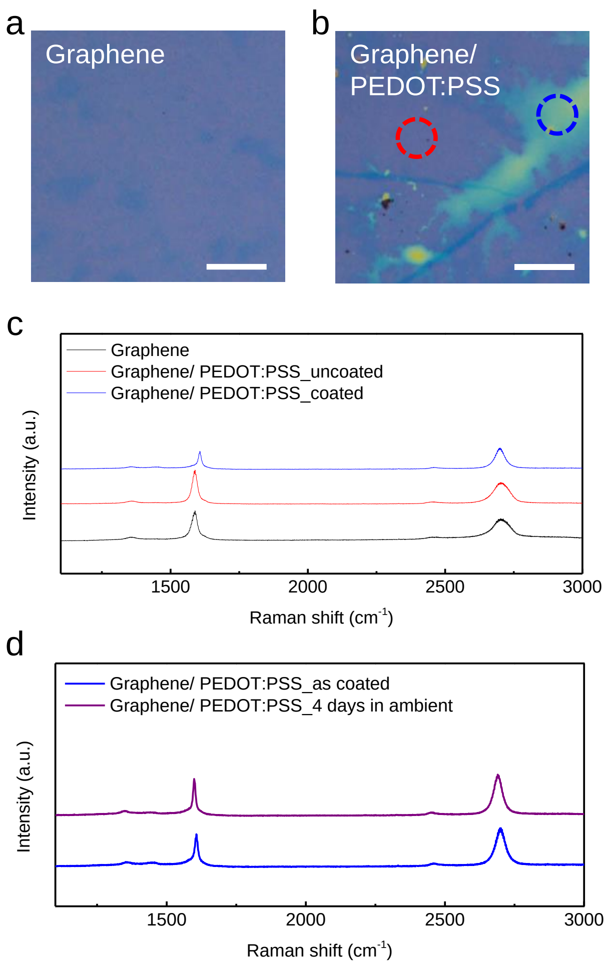

Supplementary Figure 15 | Doping characteristics of PEDOT:PSS on graphene. Optical microscopy image of (a) pristine graphene, and (b) PEDOT:PSS coated graphene. Scale bar: 100 μm. Raman spectra of (c) different regions marked in (b), and (d) PEDOT:PSS coated region and after exposure to ambient conditions for 4 days.

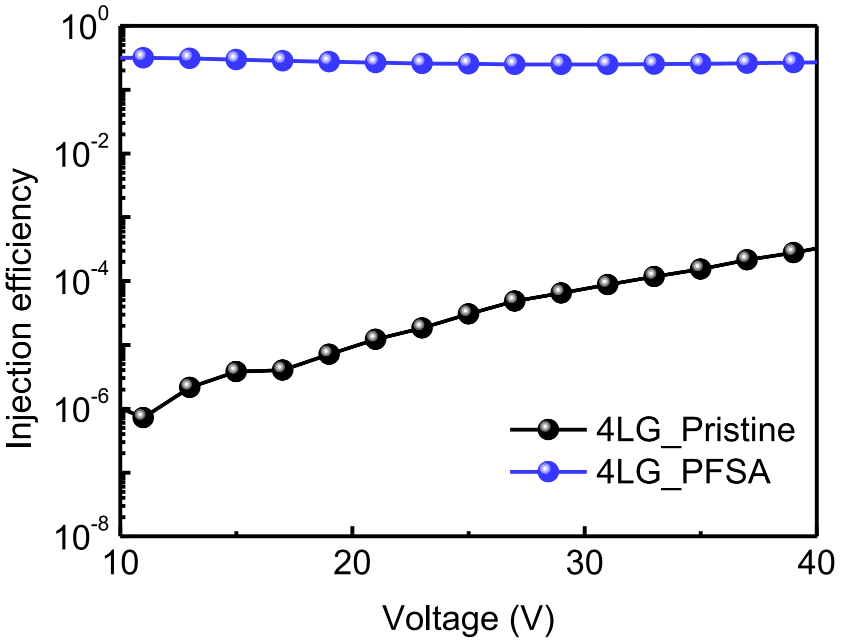

Supplementary Figure 16 | Charge injection characteristics. Charge injection efficiency of HODs with pristine and PFSA-doped graphene anode.

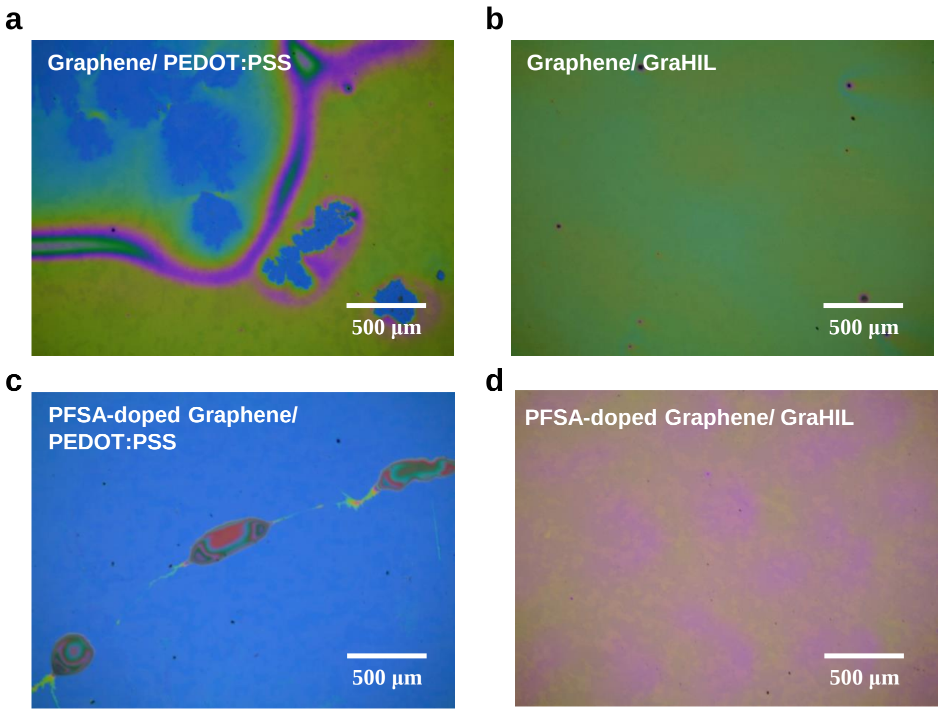

Supplementary Figure 17 | Polymeric HIL deposition on graphene anode. Optical microscopy images of (a) PEDOT:PSS on graphene, (b) GraHIL on graphene, (c) PEDOT:PSS on PFSA-doped graphene, and (d) GraHIL on PFSA-doped graphene (scale bar, 500 µm).

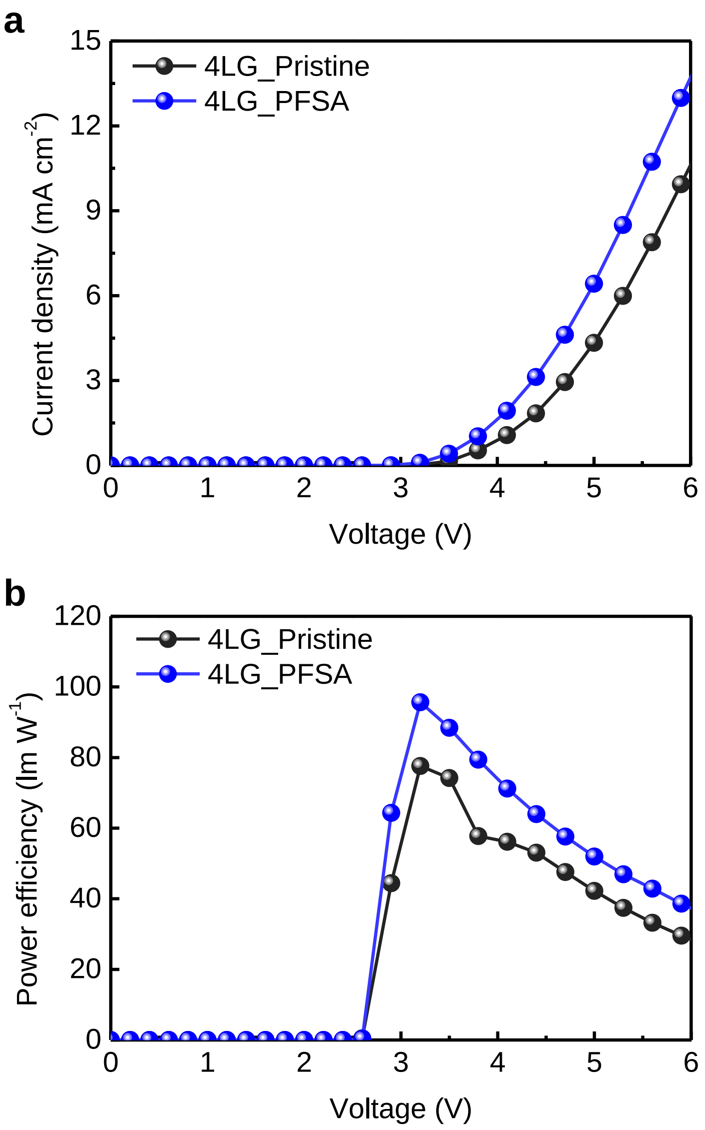

Supplementary Figure 18 | Device characteristics of OLEDs using PFSA-doped graphene anode. (**a**) Current densities, and (**b**) power efficiencies versus voltage of green phosphorescent OLEDs with pristine and PFSA-doped graphene anodes.

Supplementary Table 1 | R_{sh} change of PFSA and HNO_3-doped graphene against various annealing temperature

			Thermal treatments			
		**As doped**	**100** °C	**200** °C	**250** °C	**300** °C
**PFSA**	R_{sh} [Ω sq^{-1}]	435.1 ± 8.48	395.8 ± 37.8	365.6 ± 37.8	352.0 ± 5.49	337.6 ± 25.9
	R_{sh} increase [%]	-	-9.03	-16.0	-19.1	-22.4
**HNO_3**	R_{sh} [Ω sq^{-1}]	215.3 ± 8.49	261.3 ± 26.1	329.9 ± 32.4	381.4 ± 32.7	472.8 ± 37.2
	R_{sh} increase [%]	-	21.4	53.3	77.2	119.6

R_{sh} increase is calculated as: $(R_{sh,asD} – R_{sh})/R_{sh,asD}$. R_{sh} of pristine single-layered graphene samples that were doped with HNO_3 and PFSA was 711.5 ± 155.6 Ω sq^{-1}, and 913.2 ± 153.3 Ω sq^{-1}, respectively.

Supplementary Table 2 | R_{sh} change of PFSA and HNO_3-doped graphene against various solvent treatments

			Chemical solvent treatments			
		**As doped**	**DI water**	**IPA**	**toluene**	**DMSO**
**PFSA**	R_{sh} [Ω sq^{-1}]	372.9 ± 36.1	379.8 ± 13.7	387.0 ± 14.9	388.8 ± 14.1	388.8 ± 11.3
	R_{sh} increase [%]	-	4.75	6.75	7.25	7.25
**HNO_3**	R_{sh} [Ω sq^{-1}]	221.2 ± 5.9	423.3 ± 94.6	449.4 ± 23.0	346.7 ± 74.9	396.1 ± 15.6
	R_{sh} increase [%]	-	91.4	103.2	56.8	79.1

R_{sh} increase is calculated as: $(R_{sh,asD} – R_{sh})/R_{sh,asD}$. R_{sh} of pristine single-layered graphene samples that were doped with HNO_3 and PFSA was 711.5 ± 155.6 Ω sq^{-1}, and 807.5 ± 149.2 Ω sq^{-1}, respectively.

Supplementary Table 3 | R_{sh} change of PFSA and HNO_3-doped graphene against various acid and bases

			Acid and base treatments			
		As doped	HCl	CH_3COOH	NH_4OH	NaOH
**PFSA**	R_{sh} [Ω sq^{-1}]	361.9 ± 33.2	370.7 ± 15.8	426.0 ± 38.3	424.2 ± 46.7	629.9 ± 17.8
	R_{sh} increase [%]	-	2.4	17.7	17.2	74.1
**HNO_3**	R_{sh} [Ω sq^{-1}]	229.3 ± 15.6	381.6 ± 83.0	500.3 ± 67.2	1329 ± 131.3	-
	R_{sh} increase [%]	-	66.4	118.2	479.5	-

R_{sh} increase is calculated as: ($R_{sh,asD}$ – R_{sh})/$R_{sh,asD}$. R_{sh} of pristine single-layered graphene samples that were doped with HNO_3 and PFSA was 700.2 ± 118.8 Ω sq^{-1}, and 807.5 ± 149.2 Ω sq^{-1}, respectively.

Supplementary Table 4 | Calculated binding energy of p-type dopants and graphene

Compounds	Binding energy [eV]
HNO_3	0.33
$(CF_3)_2CF$-O-CF_2-$(CF_3)CF$-O-$CF_2CF_2SO_3H$	0.79
CF_3-(CF_2)-CF_3CF-O-CF_2-$(CF_3)CF$-O-$CF_2CF_2SO_3H$	0.95
CF_3-$(CF_2$-$CF_2)$-CF_3CF-O-CF_2-$(CF_3)CF$-O-$CF_2CF_2SO_3H$	0.99

Supplementary Note 1 | Surface morphology

Peak heights along the cross section of the PFSA-doped graphene were < 1 nm because deposition of thin polymeric layer can flatten uneven regions of the pristine graphene, such as wrinkles and grain boundaries; in contrast the peak heights on the pristine graphene were ~3 nm. All surface topographic images were measured over 5 μm × 5 μm of the graphene surface (Supplementary Fig. 1).

Supplementary Note 2 | Stability against acid and base chemicals

We performed stability test against acid to base chemicals. We dipped HNO_3- and PFSA-doped graphene into the solution of strong acid (HCl, $K_a > 1$), weak acid (CH_3COOH, K_a: ~1.8×10^{-5}), weak base (NH_4OH, K_b: ~1.8×10^{-5}), strong base (NaOH, $K_b > 1$) for 15 s, then removed the acid or base residue by blowing N_2. The surfaces of the HNO_3-doped graphene were torn by surface treatment with base chemicals (Supplementary Fig. 6a); in contrast, the surfaces of PFSA-doped graphene were much less affected even by strong base solution, and resulted in much smaller R_{sh} increase in HNO_3-doped graphene (Supplementary Fig. 6b). This result also proves the chemical stability of PFSA-doping on graphene.

Supplementary Note 3 | Surface energy calculation

Young's equation is

$$\gamma_{sl} = \gamma_s - \gamma_l cos\theta. \quad (1)$$

Considering the Owens and Wendt assumption that divides surface energy (γ_s) into dispersive ($\gamma_s{}^d$) and polar surface energy ($\gamma_s{}^p$), the following equation can be achieved:

$$\gamma_{sl} = \gamma_s + \gamma_l - 2(\gamma_s{}^d \gamma_l{}^d)^{0.5} - (\gamma_s{}^p \gamma_l{}^p)^{0.5}). \quad (2)$$

Combining Supplementary Eq.1 and Eq, 2 yields

$$({\gamma_s}^d {\gamma_l}^d)^{0.5} + ({\gamma_s}^p {\gamma_l}^p)^{0.5} = 0.5\gamma_l(1 + cos\theta). \quad (3)$$

Surface energies (γ) of graphenes were calculated by measuring contact angle (θ) of two solvents (DI water and diiodomethane), substituting the θ and constant of each solvent into Supplementary Eq.3, then solving the simultaneous equations for γ.

Supplementary Note 4 | Spatial uniformity investigation using c-AFM

We performed conductive atomic force microscopy (c-AFM) to investigate spatially resolve the electrical properties of graphene films according to doping methods (Supplementary Fig. 9). In-plane conductance of HNO_3-doped and PFSA-doped graphene were monitored after 5 days in ambient condition by measuring current between p-doped graphene and Sb-doped Si tip. p-Type doping with HNO_3 or PFSA did not affect the surface topography of graphene. PFSA-doped graphene film showed uniform current in surface mapping, the HNO_3-doped graphene surface showed significant heteroheneity of in-plane conductance. This result confirms spatial uniformity and excellent doping stability of PFSA-doped graphene.

Supplementary Note 5 | Ambient stability investigation using Raman spectroscopy

After ambient exposure for 15 days, *n* of PFSA-doped graphene did not change, but *n* of HNO_3-doped graphene decreased drastically.

Supplementary Note 6 | Ambient stability investigation using Field-Effect Transistors

Ambient stability of PFSA doping was monitored by measuring the electrical characteristics of the FETs with PFSA-doped graphene (Supplementary Fig. 11a,b). The upshift of the Dirac

point of PFSA-doped graphene did not decrease, and even slightly increased after the sample was held in ambient conditions for 21 days. Calculated average hole concentration (PFSA as-doped: 7.65×10^{12} cm^{-2}, 7 days: 9.14×10^{12} cm^{-2}, 21 days: 1.03×10^{13} cm^{-2}) also prove that PFSA doping of graphene is stable in ambient conditions (Supplementary Fig. 11c).

Supplementary Note 7 | DFT calculation using enlarged PFSA structures

DFT calculation was performed by adding fluorinated alkyl substituent ($-CF_2$ and $-C_2F_4$) to the simplest PFSA (i.e., $(CF_3)_2CF$-O-CF_2-$(CF_3)CF$-O-$CF_2CF_2SO_3H$). The most stable configurations of the simplest PFSA-CF_2 (i.e., $CF_3(CF_2)CF_3CF$-O-CF_2-$(CF_3)CF$-O-$CF_2CF_2SO_3H$) and the simplest PFSA-C_2F_4 (i.e., $CF_3(CF_2)_2CF_3CF$-O-CF_2-$(CF_3)CF$-O-$CF_2CF_2SO_3H$) and graphene were found (Supplementary Fig. 12). DFT calculation reveals that binding energy between PFSA molecules and graphene gradually increases (0.79-0.99 eV) as fluorinated alkyl substituents are added (Supplementary Table 4); this result shows that chemical doping of graphene using macromolecules would lead to high binding energy and impart excellent doping stability to graphene.

Supplementary Note 8 | X-ray photoelectron spectroscopy

We performed X-ray photoelectron spectroscopy (XPS) to investigate the change of chemical composition by PFSA doping. Pristine and PFSA-doped graphene showed an O*1s* peak that could be caused by oxygen in the glass substrate, or in PMMA left after the wet-transfer process. PFSA-doped graphene showed an intense F*1s* peak (~690 eV), and S*2p* peak (~170 eV) (Supplementary Fig. 13a). The C*1s* spectrum of PFSA-doped graphene revealed C-C sp^2 bonding (~284.7 eV), with four PFSA-related chemical bonds (i.e., C-O-C (~286.5 eV), C-S (~289.6 eV), $-CF_2$ (~292.4 eV), $-CF_3$ (~294.0 eV),[23,61] which can be confirmed in the chemical

structure of PFSA (Supplementary Fig. 13b,c); these results confirm that a PFSA layer forms on the graphene surface.

Supplementary Note 9 | Control measurements using PMMA

As a control measurement, we compared doping effect of PFSA with that of another insulating polymer, poly(methyl methacrylate) (PMMA), that is generally used as a polymer supporter during wet-transfer of graphene. We deposited ~3 nm-thick PFSA or PMMA on graphene. R_{sh} change of polymer-doped graphene was measured using 4-point probe methods, and WF change of polymer-doped graphene was measured using a SKP-5050 Kelvin Probe. Even though both polymer films had the same thickness, p-type doping was much more effective with PFSA (WF increased by ~0.8 eV, and R_{sh} decreased by ~53.3%) than with PMMA (negligible WF increase, and R_{sh} decreased by ~29.4%) (Supplementary Fig. 14c, d). In contrast to the PFSA, PMMA has neither fluorinated nor acidic groups, so it cannot induce significant p-type doping effect.

Supplementary Note 10 | Control measurements using PEDOT:PSS

PEDOT:PSS was spin-cast on pristine graphene layer without any treatment to make graphene surface hydrophilic. Film uniformity of spin-cast PEDOT:PSS on hydrophobic graphene surface was poor, and left may uncoated regions (Supplementary Fig. 15a, b). Raman spectra indicated the doping characteristics of graphene/ PEDOT:PSS. There was no clear shift of Raman characteristic bands of the uncoated region (G band of pristine graphene: 1589 cm^{-1}, G band of uncoated region: 1589 cm^{-1}). On the contrary, the coated region showed upshift of the G band to 1607 cm^{-1} (Supplementary Fig. 15c); this change indicates that PEDOT:PSS imparted p-type doping on graphene. To investigate the ambient stability of PEDOT:PSS-doped

graphene, the Raman spectrum was measured again. PEDOT:PSS-doped graphene was stored in ambient conditions for 4 days (Supplementary Fig. 15d); the upshifted G band position then downshifted from 1607 cm^{-1} to 1597 cm^{-1}; this change indicates that the p-type doping effect had weakened. The change occurs because PSS is hygroscopic, so it takes up moisture from ambient air and thereby degrades the p-type doping effect of PEDOT:PSS on graphene.

Supplementary Note 11 | Hole-injection efficiency calculation

We calculated hole-injection efficiency (η) by using theoretically-calculated space-charge limited current (SCLC) as followed by Supplementary Eq. 4.

$$J_{SCLC} = \frac{9}{8}\varepsilon\varepsilon_0\mu_0 \exp(0.89\beta\sqrt{E})\frac{E^2}{d}, \quad (4)$$

where ε = 3 is the dielectric constant of a organic materials, ε_0 = 8.85 × 10^{-14} C V^{-1} is vacuum permittivity, μ_0 = 2.5 × 10^{-4} cm^2 V^{-1} s^{-1} is zero-field carrier mobility, β = 1.2 × 10^{-3} $cm^{1/2}$ $V^{-1/2}$ is the Poole-Frenkel factor [1,2], E [V cm^{-1}] is the electric field, and d [cm] is the thickness of the organic film. η of HOD with PFSA-doped graphene was increased by > 10^3 times (Supplementary Fig. 16). The energy barrier to hole injection is substantially reduced by using PFSA-doped 4LG anode (~5.1 eV) (HOMO energy level of NPB ~5.4 eV), so hole injection to the overlying organic layer can be facilitated.

Supplementary Note 12 | Polymeric HILs deposition on PFSA-doped graphene

To deposit polymeric hole-injection layers (HILs)[3], we used perfluorinated ionomer (PFI)-blended PEDOT:PSS solution (we call it GraHIL), which contains isopropyl alcohol (IPA) rather than as-purchased PEDOT:PSS solution, because it cannot be uniformly coated on hydrophobic graphene surface (Supplementary Fig. 17a). The OLEDs were fabricated with a

GraHIL, which has high surface WF ~5.95 eV.[4-6] Diluting with IPA significantly reduced the contact angle of polymeric solution on graphene surface, because the lower polarity of IPA than that of H_2O improved the wettability. However, diluting with IPA was still insufficient to form uniform polymeric HILs on graphene surface. Blending with PFI as well as addition of IPA to PEDOT:PSS solution yielded a uniformly-cast PEDOT:PSS film on hydrophobic graphene surface. To make the graphene surface hydrophilic, we also performed mild UV-O_3 treatment before spin-casting the PFI-blended PEDOT:PSS solution. These attempts resulted in a uniform polymeric HIL film on graphene electrode (Supplementary Fig. 17b). PFSA-doped graphene has even lower surface energy (21.69 mJ m^{-2}) than pristine graphene (55.73 mJ m^{-2}) and therefore has poor film-formability (Supplementary Fig. 17c). However, improved wettability from IPA dilution, and PFI blending works in PFSA-doped graphene, so uniform polymeric HILs formed on PFSA-doped graphene surface (Supplementary Fig. 17d).

Supplementary Note 13 | Device characteristics with PFSA-doped graphene anodes

The OLED with the PFSA-doped graphene anode exhibited a higher current density than did the OLED with pristine 4LG anode; this result was also caused by improved hole injection from graphene anode due to the increased surface WF of PFSA doped graphene (Supplementary Fig. 18a). The device with PFSA-doped 4LG also showed higher power efficiency (PE) (~95.6 lm W^{-1} without an outcoupling structure) than did the device with the pristine 4LG (~77.6 lm W^{-1}) (Supplementary Fig. 18b).

Supplementary References

1. Han, T.-H. *et al*. Versatile p-type chemical doping to achieve ideal flexible graphene Electrodes. *Angew. Int. Chem. Ed*. **55**, 6197-6201 (2016).
2. Han, T.-H. *et al*. Extremely efficient flexible organic light-emitting diodes with modified graphene anode. *Nat. Photon*. **6**, 105-110 (2012).
3. Kim, H. *et al*. Organic solar cells using CVD-grown graphene electrodes. *Nanotechnology* **25**, 014012 (2014).
4. Han, T.-H., Song, W. & Lee, T.-W. Elucidating the crucial role of hole injection layer in degradation of organic light-emitting diodes. *ACS Appl. Mater. Interfaces* **7**, 3117-3125 (2015).
5. Han, T.-H. *et al*. Molecularly controlled interfacial layer strategy toward highly efficient simple-structured organic light-emitting diodes. *Adv. Mater.* **24**, 1487-1493 (2012).
6. Choi, M.-R. *et al*. Soluble self-doped conducting polymer compositions with tunable work function as hole injection/extraction layers in organic optoelectronics. *Angew. Chem. Int. Ed.* **50**, 6274-6277 (2011).
